# The Hospitals of Paris

**Published:** 1908-02-01

**Authors:** 


### THE HOSPITALS OF PARIS.

Mr. Conrad W. Thies, the Secretary of the Royal Free Hospital, read an interesting paper on this subject before the Hospital Officers' Association on the 10th ultimo. Mr. Thies has had the advantage of visiting Paris on several occasions, and has taken a great interest in the hospitals of that city. Up to 1789 all French hospitals, asylums, and charitable institutions were managed and financed by religious organisations, being at that time entirely independent of State or municipal control. In 1794 the National Assembly passed a law the effect of which was to bring all these institutions and their properties under the control of special committees appointed by the Government. The city of Paris was treated separately, and the whole of its hospitals and other institutions for the treatment of the sick and infirm, including the lunatic asylums and orphanages, are under the administrative control of a central authority appointed by the Government, and entitled "the Administration General of the Public Assistance of Paris." The Assistance combines the functions of the Metropolitan Asylum Board, together with the administration of all the hospitals and asylums of the city. Mr. Thies mentioned these facts as proofs that the French recognise that the treatment of the sick and the study of the best methods of combating diseases are the proper function of the public authorities, and should so constitute a necessary department of the State or municipality.

Under the control of the Assistance Publique of Paris there are fourteen general hospitals, containing, roughly, 8,500 beds; seven special hospitals with 2,450 beds; six maternity hospitals with 1,700 beds; and seven children's hospitals with 2,850 beds. The total number of beds is roughly about 15,500, exclusive of some thirty other institutions for the insane and inebriate, and for special classes of sick and infirm people. With a view to economy and uniformity the whole of the Paris hospitals are supplied from large central stores, which are so organised as to include provisions, dressings, and drugs, wines and spirits, coal, and in fact every article needed for each of the various institutions under the charge of the Assistance Publique. Mr. Thies alluded to the reference in the English papers to important changes in regard to the Paris hospitals, including the removal of the older ones from the centre of Paris to the outskirts of the city. He stated, as the result of his investigation, that, although, the trend of opinion is setting in this direction, there is no immediate intention to carry out any such scheme with rapidity and thoroughness. Gradually such a change may come, but it will take years before adequate expression can be given to the French desire for improved buildings and the best hygienic conditions. The authorities have, however, in hand the reconstruction of some of the hospitals in the outer ring, and they will proceed gradually to close those situated nearer the centre, which are deemed unsuitable to modern medical and surgical requirements.

Mr. Thies then proceeded to give a short summary of a few of the hospitals. The *Hôtel Dieu* occupies a position of prestige in Paris analogous to that of St. Bartholomew's Hospital in London. It is the oldest hospital in Paris, and one of the most ancient in Europe. Founded and managed by monks from the 17th century onwards, it has had a most interesting history, but the present buildings are antiquated for hospital purposes, having been designed in 1864. It contains 885 beds, divided into three departments, *i.e.*, medicine, surgery, and ophthalmology. Mr. Thies was impressed by the somewhat formal and cheerless aspect of the wards, and the absence of a trained and sympathetic nursing staff. The nursing arrangements in all Paris hospitals are inferior to those in this country. Neither the religious sisters nor the lay sisters impressed Mr. Thies with the feeling that they were under discipline or drawn from a sufficiently high social grade. Mr. Thies observed a general want of attention to details, an air of slovenliness, and sometimes a lack of perfect cleanliness.

The *Charité*, with 654 beds, was founded in 1613, and is worked by the Brethren of St. Jean de Dieu. These monks were trained as surgeons and nurses, and were famous in their day as successful operators. This caused jealousy to arise in the College of Surgeons, who proceeded against them by law on the ground that they taught the art of surgery, a function which by its charter was the monopoly of their College. This hospital is not up to the present standard of medical and surgical science, and will probably soon disappear.

*La Pitié* is a very old hospital with 680 beds. It is also condemned. The *St. Antoine* contains 865 beds; the *Necker* 478; the *Cochin* 809; and the *Beaujon* 608 beds. All these were founded between 1778 and 1795. The *Lariboisière*, erected in 1852, is a large and comparatively modern institution with one thousand beds. It has a memorable history, having been bombarded for two days during the Commune in 1871, but the surgeons and their assistants remained at their posts both night and day. In those two days three hundred dead or wounded were received into the hospital. It is at present the most popular hospital in Paris: every class of disease is treated there, and it is always crowded with patients.

The *Boucicaut Hospital* was built in 1897, and contains 300 beds. This is the most modern of all the Paris hospitals, the arrangements for heating, lighting, and ventilation being well up to date. Each patient has a bath before admission; every particle of clothing is sent to the sterilising room, and each patient is supplied with fresh clothing, provided by the hospital. It contains most elaborate provision for gynæcological and maternity cases.

The *Britonneau*, with 266 beds, is the principal children's hospital. The modern buildings exhibit taste and elegance. The walls of some of the wards are decorated in coloured glass mosaics, representing some of Æsop's fables, and Mr. Thies consider this an ideal children's hospital. The *Hertford British Hospital*, with forty beds, was opened in 1879. It was founded and endowed by Sir Richard and Lady Wallace; and Miss Davidson, the matron, is always most willing to welcome English visitors. Unfortunately those responsible for the planning appear not to have been conversant with hospital requirements, which have been sacrificed to architectural effect. The internal administration is good, and the wards are well kept and spotlessly clean, but there are very few patients. The hospital requires reorganisation throughout to bring it on to better and more workable lines.

Mr. Thies concluded his paper with a brief reference to the Pasteur Institute, which cost £150,000, provided by public subscriptions and by the late Baroness de Hirsch. Each visitor before entering this hospital has to don a long white cotton over-all with a cap to match. The patients are treated in separate compartments, the lower portion of which to the height of about five feet is constructed of metal, and the entire upper part of glass. The most minute precautions are taken to prevent the spread of infection, and the patients are kept under constant observation day and night. Mr. Thies stated for the encouragement of English visitors who are interested in hospitals that there is no difficulty in obtaining the necessary permit to visit the hospitals. A personal application at the offices of the Assistance Publique, 3 Avenue Victoria, will produce an order of admission, on presentation of which at any hospital some one will be instructed to take the visitor through the institution.

